# Neonatal mortality in NHS maternity units by timing of birth and method of delivery: a retrospective linked cohort study.

**DOI:** 10.23889/ijpds.v7i3.2024

**Published:** 2022-08-25

**Authors:** Lucy Carty, Mario Cortina-Borja, Rachel Plachcinsk, Chris Grollman, Alison Macfarlane

**Affiliations:** 1 UK Health Security Agency; 2 UCL Great Ormond Street Institute of Child Health; 3 School of Health Sciences, City, University of London; 4 Nuffield Dept. of Women's and Reproductive Health, Oxford

## Objectives

Potential ‘weekend effects’ in healthcare prompt concerns that care could be of lower quality during non-working hours, but may reflect differences in case mix or other factors This research aimed to compare neonatal mortality in English hospitals from 2005 to 2014 by time of day and day of the week.

## Approach

We analysed data from a retrospective cohort of 6,054,536 singleton births in England 2005—2014, created by linking ONS birth and death registration and birth notification data with Hospital Episode Statistics.

Working hours were defined as 07:00—19:00 on weekdays, and non-working hours were all other times on weekdays and all weekends and public holidays.

The primary outcome was all-cause neonatal mortality unattributed to congenital anomaly. We also modelled cause-specific neonatal mortality attributed to asphyxia, anoxia or trauma (AAT). On advice through our public involvement and strategy, analysis was stratified by mode of onset of labour and method of delivery.

## Results

After adjustment for confounders, the odds of all-cause neonatal mortality outside of working hours were similar to those during working hours for spontaneous births, instrumental births and emergency caesareans. Planned caesareans occurring in non-working hours had a high crude risk compared to planned caesareans in working hours, but were considered to be unreliably recorded and likely to reflect emergency caesarean delivery of babies originally scheduled for planned caesarean birth.

Further stratification of emergency caesareans by onset of labour showed higher odds of cause-specific neonatal mortality (AAT) during non-working compared with working hours for emergency caesareans without labour recorded but not for emergency caesareans after spontaneous or induced onset of labour.

## Conclusion

It may be that the apparent ‘weekend effect’ is caused by deaths among the relatively small number of babies who were born by caesarean section apparently without labour outside normal working hours. Obstetric staffing should be planned to allow for these relatively unusual emergencies.

